# MicroRNA-146a-5p Mediates High Glucose-Induced Endothelial Inflammation via Targeting Interleukin-1 Receptor-Associated Kinase 1 Expression

**DOI:** 10.3389/fphys.2017.00551

**Published:** 2017-08-02

**Authors:** Wan-Yu Lo, Ching-Tien Peng, Huang-Joe Wang

**Affiliations:** ^1^Cardiovascular & Translational Medicine Laboratory, Department of Biotechnology, Hungkuang University Taichung, Taiwan; ^2^Department of Pediatrics, Children's Hospital, China Medical University and Hospital Taichung, Taiwan; ^3^Department of Biotechnology, Asia University Taichung, Taiwan; ^4^School of Medicine, China Medical University Taichung, Taiwan; ^5^Division of Cardiovascular Medicine, Department of Medicine, China Medical University and Hospital Taichung, Taiwan; ^6^Cardiovascular Research Laboratory, China Medical University and Hospital Taichung, Taiwan

**Keywords:** diabetes, high glucose, endothelial inflammation, Interleukin-1 receptor-associated kinase-1, miR-146a-5p

## Abstract

**Background and Aims:** Interleukin-1 receptor-associated kinase-1 (IRAK-1) is critical for mediating toll-like receptor and interleukin-1 receptor signaling. In this study, we have examined whether IRAK-1 expression is altered in high glucose (HG)-stimulated human aortic endothelial cells (HAECs), and whether microRNAs (miRs) target IRAK-1 to regulate HG-induced endothelial inflammation.

**Methods:** HAECs were treated with HG for 24 and 48 h. Real-time PCR, Western blot, monocyte adhesion assay, bioinformatics analysis, TaqMan® arrays, microRNA mimic or inhibitor transfection, luciferase reporter assay and siRNA IRAK-1 transfection were performed. The aortic tissues from db/db type 2 diabetic mice were examined by immunohistochemistry staining.

**Results:** HG time-dependently increased IRAK-1 mRNA and protein levels in HAECs, and was associated with increased VCAM-1/ICAM-1 gene expression and monocyte adhesion. Bioinformatic analysis, TaqMan® arrays, and real-time PCR were used to confirm that miR-146a-5p, miR-339-5p, and miR-874-3p were significantly downregulated in HG-stimulated HAECs, suggesting impaired feedback restraints on HG-induced endothelial inflammation via IRAK-1. However, only miR-146a-5p mimic transfection reduced the HG-induced upregulation of IRAK-1 expression, VCAM-1/ICAM-1 expression, and monocyte adhesion. Additionally, IRAK-1 depletion reduced HG-induced VCAM-1/ICAM-1 gene expression, and monocyte adhesion, indicating that HG-induced endothelial inflammation was mediated partially through IRAK-1. *In vivo*, intravenous injections of miR-146a-5p mimic prevented endothelial IRAK-1 and ICAM-1 expression in db/db mice.

**Conclusion:** These results suggest that miR-146a-5p is involved in the regulation of HG-induced endothelial inflammation via modulation of IRAK-1; indicating that miR-146a-5p may be a novel target for the treatment of diabetic vascular complications.

## Introduction

The increasing number of people with obesity, advanced age, and physically inactive lifestyles contributes to the increased incidence of diabetes, which had an estimated global prevalence of 6.4% in 2010 (Shaw et al., 2010). Diabetic vascular disease is a chronic inflammatory disease that accounts for the majority of morbidity and mortality in diabetic patients. Hyperglycemia in diabetes causes endothelial dysfunction (Nakagami et al., 2005), a condition characterized by impaired vasodilatation, proinflammation, prothrombosis, and impaired endothelial repairs that precipitate atherosclerotic progression and atherothrombotic complications (Sena et al., 2013).

Interleukin-1 receptor activated kinases (IRAKs) are the key mediators of innate immunity. The mammalian IRAK family consists of four members: IRAK-1, IRAK-2, IRAK-M, and IRAK-4. Of these, IRAK-1 was the first to be identified; its role as an adaptor and kinase is essential for the toll-like receptor (TLR) and interleukin-1 receptor (IL-1R) signaling pathways, which regulate cellular inflammation (Gottipati et al., 2008). TLR signaling is initiated by ligand-induced dimerization of receptors (e.g., lipopolysaccharide ligand (LPS)–TLR4 receptors). Following the recruitment of adaptor molecules, including myeloid differentiation primary response protein (MYD88), the downstream signaling pathways involve the interaction of interleukin-1 receptor-associated kinase 1 (IRAK-1) and TNF receptor-associated factor 6 (TRAF-6) (Flannery and Bowie, 2010). Downstream from TRAF-6, transcription factors, including nuclear factor κB (NF-κB), interferon-regulatory factor 5, cyclic AMP response element-binding protein, and activator protein 1, are activated to induce the production of inflammatory cytokines and type 1 interferon (O'Neill et al., 2013; Jain et al., 2014). In addition, IRAK-1 signaling can be initiated through IL-1R upon ligand binding (Jain et al., 2014). IL-1R activation can drive a variety of inflammatory mediators (e.g., interleukin 6, tumor necrosis factor α) to induce host protection (Gottipati et al., 2008). Hyperglycemia has been suggested to cause dysregulated innate immunity (Jafar et al., 2016; Kousathana et al., 2017). In addition, innate immunity-induced inflammation is important for the pathogenesis and disease progression of both type 1 and type 2 diabetes (Prajapati et al., 2014; Cabrera et al., 2016; Wada and Makino, 2016; Mistry et al., 2017). However, little is known about the role of IRAK-1 in diabetes.

MicroRNAs (miRs) are important post-transcriptional regulators of the endothelial oxidative and inflammatory responses (Marin et al., 2013). Increasing evidence suggests that miRs are involved in the pathogenesis of diabetes (Guay et al., 2011). Additionally, diabetic complications can be predicted from the circulating levels of certain miRs (Guay and Regazzi, 2013). Although utilizing differential miRs as therapeutic targets in diabetes is a potentially promising strategy, the human genome encodes more than 1,600 miR precursors, making the identification of potential miR targets difficult (Kolfschoten et al., 2009; Guay and Regazzi, 2013).

In this study, we have investigated the potential miRs that regulate IRAK-1 expression in high glucose (HG)-stimulated human aortic endothelial cells (HAECs), and performed an *in vivo* examination of aortic endothelial cells from db/db type 2 diabetic mice.

## Materials and methods

### Cell culture

HAECs were purchased from Cell Applications, Inc. (San Diego, CA, USA) and cultured in endothelial cell growth medium (Cell Applications, Inc.) according to the manufacturer's recommendations. All chemicals were obtained from Sigma-Aldrich (St. Louis, MO, USA) unless otherwise specified. High glucose (HG, 25 mM) was added to HAECs for 24 and 48 h in the different experiments. Mannitol (25 mM) was the osmotic control. The human monocytic cell line THP-1 was obtained from the American Type Culture Collection (Rockville, MD, USA), and maintained in RPMI 1640 culture medium supplemented with 10% FBS, L-glutamine, and penicillin.

### Real-time polymerase chain reaction (PCR)

Expression of mRNA in the HAECs was analyzed by real-time PCR as previously described (Wang et al., 2010). The primer sequences for glyceraldehyde-3-phosphate dehydrogenase (GAPDH), vascular cell adhesion protein 1 (VCAM-1), and intercellular adhesion molecule 1 (ICAM-1) are provided in Supplemental Data 1.

### Western blot analysis

Protein expression levels in the HAECs were analyzed by western blot as previously described (Wang et al., 2010). Antibodies against IRAK-1 (Cell Signaling Technology, Danvers, MA, USA), and GAPDH (Santa Cruz Biotechnology, Santa Cruz, CA, USA) were used at 1:1,000.

### Monocyte adhesion assay

In adhesion experiments, THP-1 cells were labeled with calcein acetoxymethyl ester (Calcein-AM; Molecular Probes, Eugene, OR, USA) as previously described (Wang, H. J. et al., 2014). Briefly, THP-1 cells were stained with the dye at a concentration of 7.5 μM for 30 min immediately preceding the adhesion assay. HAECs were maintained in 12 well-plates until 90% confluence. The HAECs (10^5^ cells/well) were then treated with HG for 24 and 48 h and incubated with culture medium containing the labeled THP-1 cells (THP-1/HAECs = 7) for 10 min. Non-adherent THP-1 cells were removed by washing with PBS for 20 s. Adherent THP-1 cells were visualized and quantified in 10 randomly viewed fields by the fluorescent microscope (OLYMPUS, Japan).

### TaqMan® Array Human MicroRNA Card analysis

The TaqMan® Array Human MicroRNA A Card V2 (Applied Biosystems, Foster, CA, USA) was used to analyze miR expression profiles. The card contains 377 preloaded human miR targets and four endogenous controls. For each sample, 500 ng of total RNA was used for reverse-transcription, using Megaplex RT primer Pool A and a TaqMan MicroRNA Reverse Transcription Kit (Applied Biosystems). The resulting cDNA was diluted, mixed with TaqMan Gene Expression Master Mix (Applied Biosystems), and loaded into the ports on microfluidic cards. The cards were briefly centrifuged for 1 min at 1,600 × *g* to distribute samples to the multiple wells, sealed to prevent well-to-well contamination, and analyzed using a 7900 HT Real-Time PCR System (Applied Biosystems).

### Extraction and analysis of HAEC miRs

The protocols for miR extraction and determination of different miR expression levels from HAECs were as previously described (Wang et al., 2013). The reverse transcription and PCR primer sequences are provided in Supplemental Data 1.

### Transfection of miR mimics and inhibitors

Selected miR mimics, inhibitors, and a negative control (NC) were transfected into HAECs as previously described (Wang, H. J. et al., 2014). After transfection, HAECs were treated with HG for 48 h, after which the expression levels of IRAK-1 mRNA, IRAK-1 protein, VCAM-1 mRNA, and ICAM-1 mRNA were determined. THP-1 adhesion assays were also performed after miR-146a-5p mimic transfection.

### Luciferase reporter assay

A partial IRAK-1 mRNA 3′-UTR containing the miR-146a-5p target site was constructed into a pGL-3-promoter vector (Promega, Madison, WI). HAECs were cotransfected with 1 μg of constructed plasmids and 100 nM of miR-146a-5p mimic and the negative control using Lipofectamine™ 2000 (Invitrogen, Carlsbad, CA). Empty vector was used as blank control. After 24 h of transfection, cells were harvested to measure luciferase activity using the Luciferase Assay System Kit (Promega, E1500), according to the manufacturer's instructions.

### IRAK-1 gene silencing

The HAECs were transfected with 100 nM of either ON-TARGETplus SMARTpool Human IRAK-1 small interfering RNA (siRNA; Dharmacon, Thermo Scientific, Lafayette, CO, USA) or a negative control, as previously described (Wang et al., 2016). The IRAK-1 siRNA target sequences were shown in Supplemental Data 2. Briefly, HAECs were transfected using Lipofectamine™ 2000 transfection reagent (Invitrogen, Carlsbad, CA, USA) in M-199 medium for 2 h. After transfection, the medium was changed to endothelial cell growth medium, and the HAECs were treated with HG for 48 h. After HG treatment, expression of IRAK-1 mRNA, IRAK-1 protein, VCAM-1 mRNA, and ICAM-1 mRNA were determined. THP-1 adhesion assays were also performed after IRAK-1 siRNA transfection.

### Type 2 diabetic mouse model studies

The animal study was conducted with the ethical standards of the field and performed in accordance with the ethical guidelines provided by the Hungkuang University Institutional Animal Care and Use Committee (Permit Number: HK 105-02). Male db/db diabetic mice were purchased from National Laboratory Animal Center (Nangang, Taipei, Taiwan). Eleven-week db/db mice were administered (100 μL) miR-146a-5p mimic or a negative control (13 μg per week, 3 times) by tail-vein injection, using equal volume mixtures of Lipofectamine™ 2000 and miR-146a-5p mimic or negative control. The control db/db group received equal volume mixtures of vehicle (Lipofectamine™ 2000) and PBS. Three weeks later, mice were euthanized by CO_2_ narcosis. The aortic tissue were carefully excised and fixed with 10% formalin solution. Paraffin sections (5 μm thickness) of aorta were prepared for immunohistochemistry (IHC) staining.

### IHC staining

For aortic tissue sections from db/db mice, 3,3′-diaminobenzidine staining was performed using a Bond-Max autostainer (Leica Microsystems). Briefly, paraffin-embedded aortic tissue sections were placed in Tris buffered saline with Tween-20, then rehydrated through serial dilutions of alcohol, and washed with PBS (pH 7.2). Slides were then stained with primary antibodies against IRAK-1 (dilution 1:50, mouse monoclonal antibody, Santa Cruz), or ICAM-1 (dilution 1:50, mouse monoclonal antibody, Thermo Fisher), or incubated with PBS (as a negative control) on a fully automated Bond-Max system using onboard heat-induced antigen retrieval and a VBS Refine polymer detection system (Leica Microsystems).

### Statistical analysis

Statistical analysis was performed using the SPSS 12.0 statistical software package for Windows (SPSS Inc., Chicago, IL, USA). All data are presented as the mean ± SEM. Independent experiments were performed to evaluate significant differences between the control and other experimental groups. Significant differences were determined using one-way analysis of variance (ANOVA) with post-hoc Tukey test or Student's *t*-tests, where appropriate. Significant differences were defined as *p* < 0.05.

## Results

### HG induced endothelial IRAK-1 expression and inflammatory phenotypes

**Figure 1:**
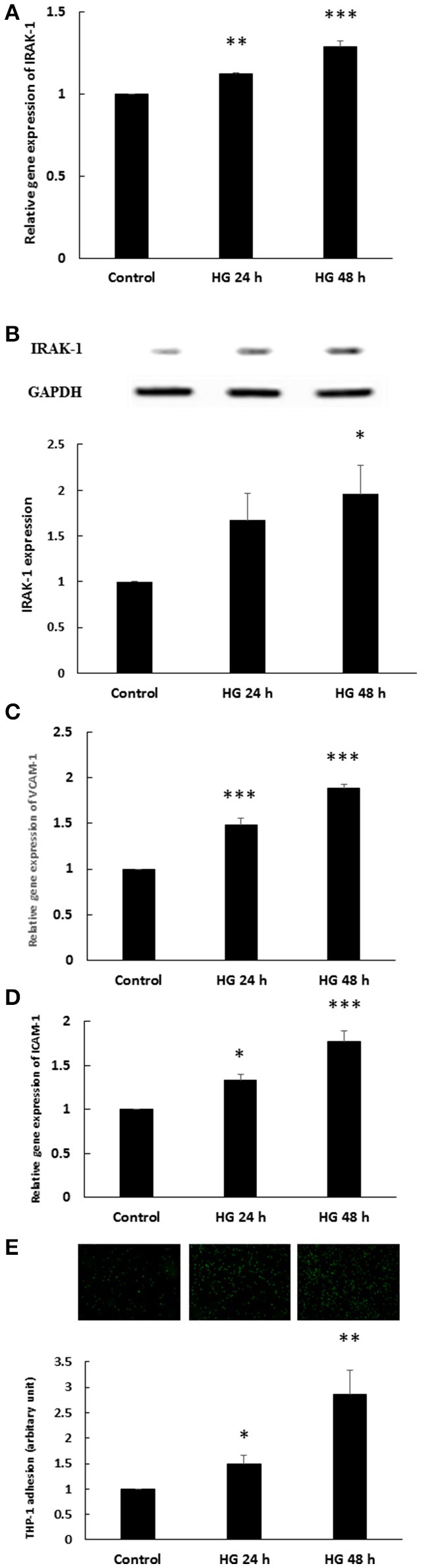
**(A–E)** Endothelial IRAK-1 expression and inflammatory phenotypes were induced by high glucose (HG). **(A)** HAECs were stimulated with HG (25 mmol/L) for 24 and 48 h. Real-time PCR demonstrates that stimulation of HAECs with HG induced 1.12- and 1.29-fold increases in IRAK-1 mRNA levels. *n* = 5. ^**^p < 0.01 and ^***^p < 0.001, compared with the control (ANOVA). **(B)** Stimulation of HAECs with HG for 24 and 48 h induced 1.67- and 1.96-fold increases, respectively, in IRAK-1 protein expression. *n* = 5, ^*^p < 0.05, compared with the control (ANOVA). **(C)** Stimulation of HAECs with HG for 24 and 48 h induced 1.48- and 1.88-fold increases, respectively, in VCAM-1 gene expression levels. *n* = 5. ^***^p < 0.001, compared with the control (ANOVA). **(D)** Stimulation of HAECs with HG for 24 and 48 h induced 1.33- and 1.77-fold increases, respectively, in ICAM-1 gene expression levels. *n* = 5. ^*^p < 0.05 and ^***^p < 0.001, compared with the control (ANOVA). **(E)** Stimulation of HAECs with HG for 24 and 48 h induced 1.50- and 2.86-fold increases, respectively, in THP-1 adhesion to HAECs. *n* = 6. ^*^p < 0.05 and ^**^p < 0.01, compared with the control (ANOVA).

We first determined the effects of HG on endothelial IRAK-1 expression. After 24 and 48 h stimulation, HG caused significant (1.12- and 1.29-fold) increases in IRAK-1 gene expression in HEACs, compared to the unstimulated control (Figure 1A). The expression of IRAK-1 protein also displayed a time-dependent increase, with 1.67- and 1.96-fold increases after 24 and 48 h of HG stimulation, respectively (Figure 1B). The osmotic control experiments showed that mannitol treatment did not modulate the expression levels of IRAK-1 (Supplemental Data 3). The adhesion of monocytes to the inflamed endothelium is a hallmark of the initiation of atherosclerotic plaques (Tuttolomondo et al., 2012). Vascular cell adhesion protein 1 (VCAM-1) and intercellular adhesion molecule 1 (ICAM-1) are essential molecules for this adhesive process. As shown in Figure 1C, HG stimulation for 24 and 48 h caused 1.48- and 1.88-fold increases of VCAM-1 gene expression, respectively. Similarly, HG caused 1.34- and 1.77-fold increases in ICAM-1 gene expression (Figure 1D). The increases of VCAM-1 and ICAM-1 expression levels were associated with increased adhesion of THP-1 monocytic cells to HAECs, as HG stimulation for 24 and 48 h caused 1.50- and 2.86-fold increases in THP-1 adhesion to HAECs, respectively (Figure 1E).

### MiR-146a-5p, miR-339-5p, and miR-874-3p were downregulated in HG-treated HAECs

**Figure 2:**
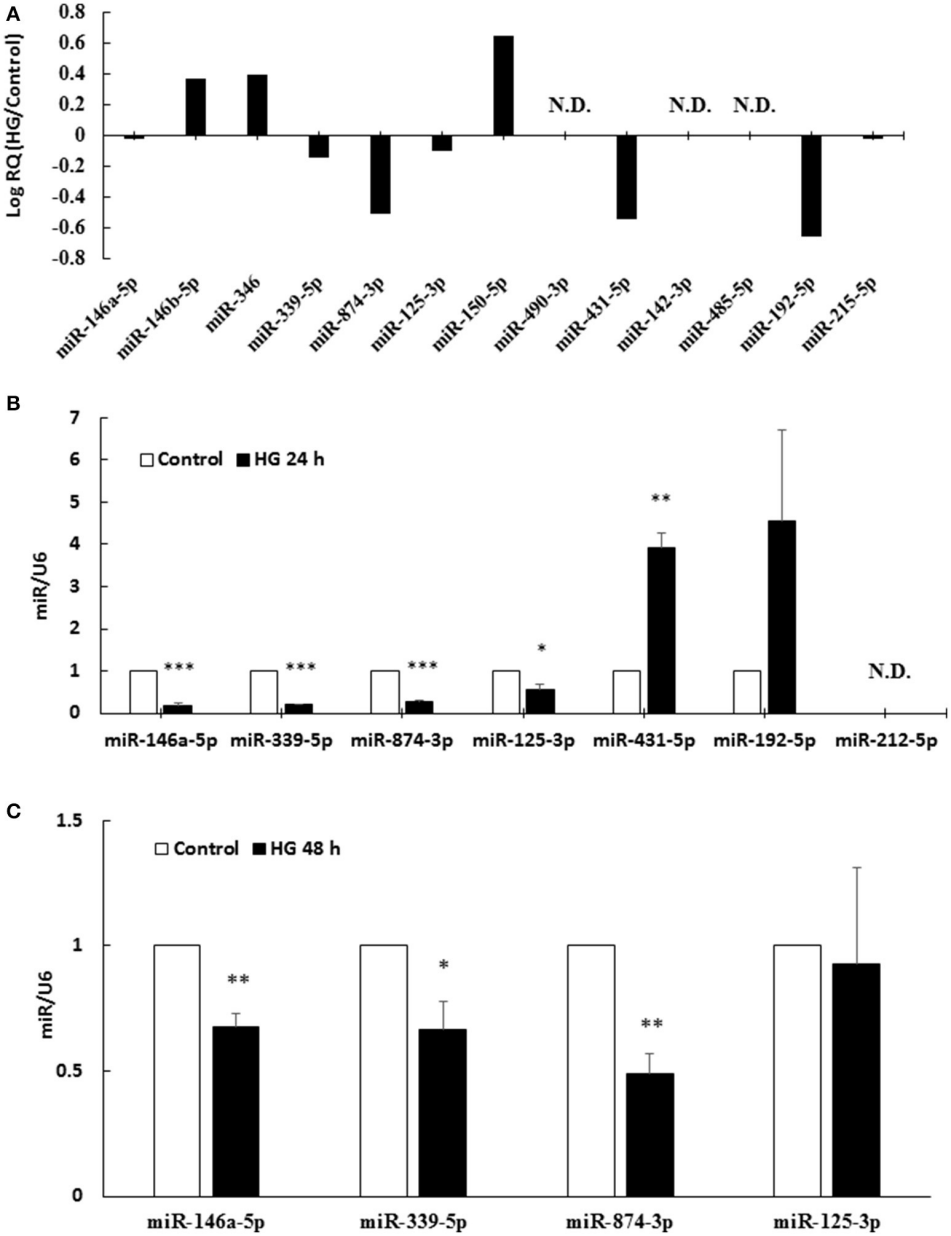
**(A–C)** MiR-146a-5p, miR-339-5p, and miR-874-3p were downregulated in 24 and 48 h HG-treated HAECs. **(A)** The TaqMan® Array Human MicroRNA Card contained all 13 possible miRs targeting IRAK-1. From left to right, the 13 miRs are ordered according to their absolute value of mirSVR score. HG stimulation for 24 h revealed that seven miRs, miR-146a-5p, miR-339-5p, miR-874-3p, miR-125-3p, miR-431-5p, miR-192-5p, and miR-215-5p, were downregulated by HG, as compared with unstimulated control. The 13 miR expression levels are expressed as log Relative Quantity (HG/control). N.D., not detected. **(B)** Seven downregulated miRs found via TaqMan® Array were confirmed by real-time PCR. HG stimulation for 24 h decreased miR-146a-5p, miR-339-5p, miR-874-3p, and miR-125-3p expression levels to 18, 20, 28, and 54% of the control level, respectively. *n* = 3. ^*^p < 0.05, ^**^p < 0.01, and ^***^p < 0.001, compared with the control (*t*-test). The levels of the miRs are expressed as the ratios of U6 levels. **(C)** Four downregulated miRs, as shown by 24 h real-time PCR, were further examined in the 48 h experiment. HG stimulation for 48 h decreased miR-146a-5p, miR-339-5p, and miR-874-3p to 68, 60, and 49% of the control level, respectively. *n* = 3. ^*^p < 0.05 and ^**^p < 0.01, compared with the control (*t*-test). The levels of the miRs are expressed as the ratios of U6 levels.

To determine potential binding partners of the 3′-UTR of human IRAK-1 mRNA, *in silico* analyses using miRanda-mirSVR (http://www.microrna.org) were performed. This online database incorporated a mirSVR scoring system to improve predictions of the effects that a miR may have on gene expression (Betel et al., 2010). A total of 13 miRs were predicted to possess homology with the 3′-UTR of the human IRAK-1 mRNA (Supplemental Data 4). The TaqMan® Array Human MicroRNA Card contained all 13 possible miRs predicted to target IRAK-1, and 24 h HG-stimulation caused the downregulation of seven endothelial miRs: miR-146a-5p, miR-339-5p, miR-874-3p, miR-125-3p, miR-431-5p, miR-192-5p, and miR-215-5p (Figure 2A). Real-time PCR analyses of 24 h HG-stimulated HAECs showed that only miR-146a-5p, miR-339-5p, miR-874-3p, and miR-125-3p expression were significantly downregulated compared to the unstimulated control (Figure 2B). These four miRs were further selected for 48 h HG stimulation experiments. When HAECs were stimulated with HG for 48 h, the expression levels of miR-146a-5p, miR-339-5p, and miR-874-3p were significantly downregulated compared to the unstimulated control (Figure 2C).

### MiR-146a-5p mimic inhibited HG-induced endothelial IRAK-1 expression and inflammatory phenotypes

**Figure 3:**
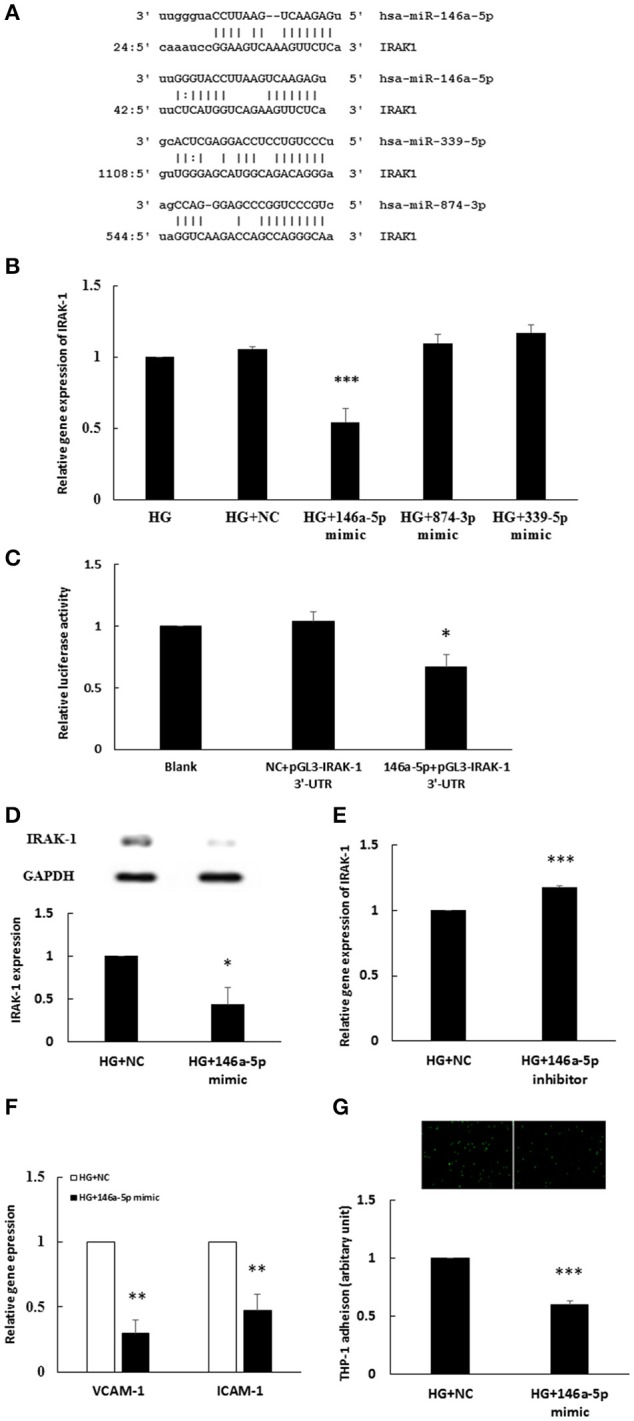
**(A–G)** The miR-146a-5p mimic inhibited HG-induced endothelial IRAK-1 expression and inflammatory phenotypes. **(A)** Bioinformatic miR target analysis identified homologies between miR-146a-5p (two binding sites), miR-339-5p, miR-874-3p, and the 3′-UTR of human IRAK-1 mRNA, indicating potential regulation of IRAK-1 by these three miRs. **(B)** Three miR mimic competition assays determined that the stimulatory effect of HG on IRAK-1 mRNA expression was inhibited only in miR-146a-5p mimic-transfected HAECs. *n* = 4. ^***^p < 0.001, as compared with HG treatment transfected with a negative control (HG+NC) (ANOVA). **(C)** A luciferase reporter assay showed that the miR-146a-5p mimic could downregulate the relative luciferase activity of pGL3-IRAK-1 3′-UTR. *n* = 5, ^*^p < 0.05, compared with the negative control (NC) (ANOVA). **(D)** The stimulatory effect of HG on IRAK-1 protein expression was inhibited in miR-146a-5p mimic-transfected HAECs. *n* = 4. ^**^p < 0.01, compared with the NC (*t*-test). **(E)** The stimulatory effect of HG on IRAK gene expression was enhanced by the miR-146a-5p inhibitor. *n* = 3. ^***^p < 0.001, compared with the NC (*t*-test). **(F)** The stimulatory effects of HG on the gene expression of VCAM-1 and ICAM-1 was inhibited in miR-146a-5p mimic-transfected, HG-stimulated HAECs. *n* = 3. ^**^p < 0.01, as compared with the NC (*t*-test). **(G)** The stimulatory effect of HG on THP-1 adhesion to HAECs was inhibited in miR-146a-5p mimic-transfected, HG-stimulated HAECs. *n* = 3. ^***^p < 0.001, as compared with the NC (*t*-test).

The miR target analysis showed homologies between the 3′-UTR of the human IRAK-1 mRNA and miR-146a-5p (two binding sites), miR-339-5p, and miR-874-3p, indicating potential regulation of IRAK-1 (Figure 3A). We performed a miR mimic competitive transfection assay to determine whether mimics of these three miRs could decrease IRAK-1 gene expression. As shown in Figure 3B, only miR-146a-5p transfection significantly decreased IRAK-1 gene expression in the 48 h HG-stimulated HAECs. The osmotic control experiments showed that mannitol treatment did not modulate the expression levels of miR-146a-5p (Supplemental Data 3). To investigate whether miR-146a-5p can interact with the IRAK-1 mRNA 3′-UTR, a luciferase reporter assay was performed. As shown in Figure 3C, cotransfection of pGL3-IRAK-1-3′-UTR and the miR-146a-5p mimic resulted in a decrease in luciferase signal to 65% of that in the negative control, which confirmed direct binding of miR-146a-5p to the IRAK-1 3′-UTR. Transfection of the miR-146a-5p mimic also significantly attenuated IRAK-1 protein expression in HG-stimulated HAECs (Figure 3D). By contrast, the stimulatory effects of HG on IRAK-1 expression were potentiated by a miR-146a-5p inhibitor (Figure 3E). To determine whether reduced IRAK-1 expression was associated with reduced endothelial inflammation in miR-146a-5p mimic-transfected, HG-stimulated HAECs, we measured VCAM-1 and ICAM-1 expression levels and THP-1 adhesion. As anticipated, both VCAM-1 and ICAM-1 levels were attenuated by transfection of the miR-146a mimic (Figure 3F). Furthermore, THP-1 adhesion to HAECs was also significantly reduced (Figure 3G).

### IRAK-1 siRNA depletion inhibited HG-induced endothelial inflammation

**Figure 4:**
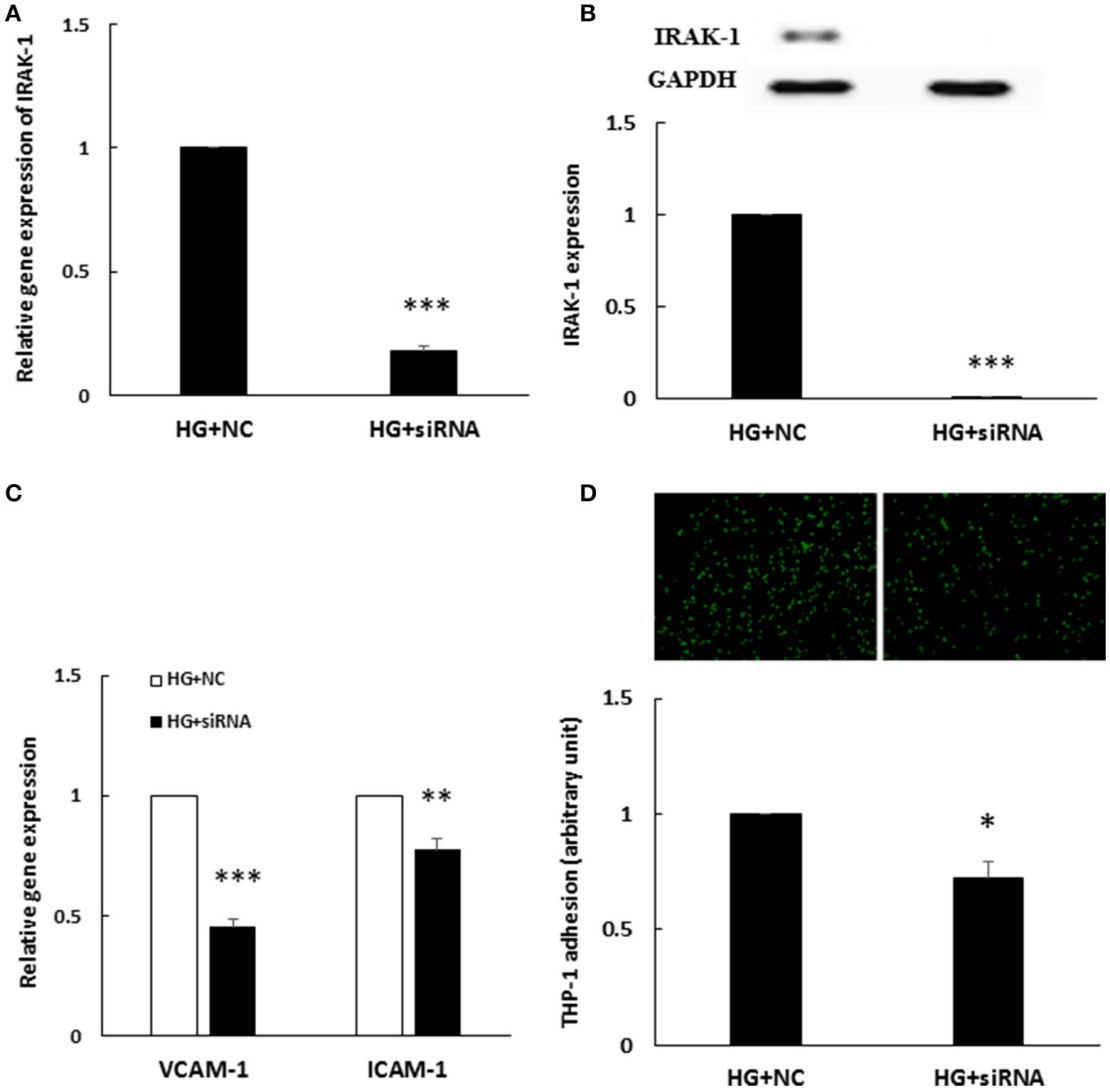
**(A–D)** HG-induced endothelial inflammation was reduced by IRAK-1 siRNA transfection **(A)**. IRAK-1 gene expression levels were reduced by IRAK-1 siRNA. *n* = 3. ^***^p < 0.001, compared with the negative control (NC) (*t*-test). **(B)**. IRAK-1 protein expression levels were reduced by IRAK-1 siRNA. *n* = 3. ^***^p < 0.001, compared with the NC (*t*-test). **(C)** The stimulatory effect of HG on VCAM-1/ICAM-1 gene expression was inhibited in IRAK-1 siRNA-transfected HAECs. *n* = 3, ^**^p < 0.01 and ^***^p < 0.001, compared with the NC (*t*-test). **(D)** The stimulatory effect of HG on THP-1 adhesion to HAECs was inhibited in siRNA IRAK-1-transfected HAECs. *n* = 3. ^*^p < 0.05, compared with the NC (*t*-test).

To determine whether enhanced IRAK-1 expression was associated with HG-induced endothelial inflammation, an IRAK-1 siRNA transfection experiment was performed. After IRAK-1 siRNA transfection, both IRAK-1 mRNA (Figure 4A) and protein levels (Figure 4B) were significantly downregulated in HG-stimulated HAECs. Furthermore, similar to the effects of the miR-146a mimic, IRAK-1 siRNA significantly inhibited the expression of HG-stimulated VCAM-1/ICAM-1 gene expression (Figure 4C) and THP-1 adhesion to HAECs (Figure 4D), indicating that HG-induced endothelial inflammation was mediated partially through IRAK-1.

### The miR-146a-5p mimic decreased endothelial IRAK-1 and ICAM-1 expression in type 2 diabetic mice

**Figure 5:**
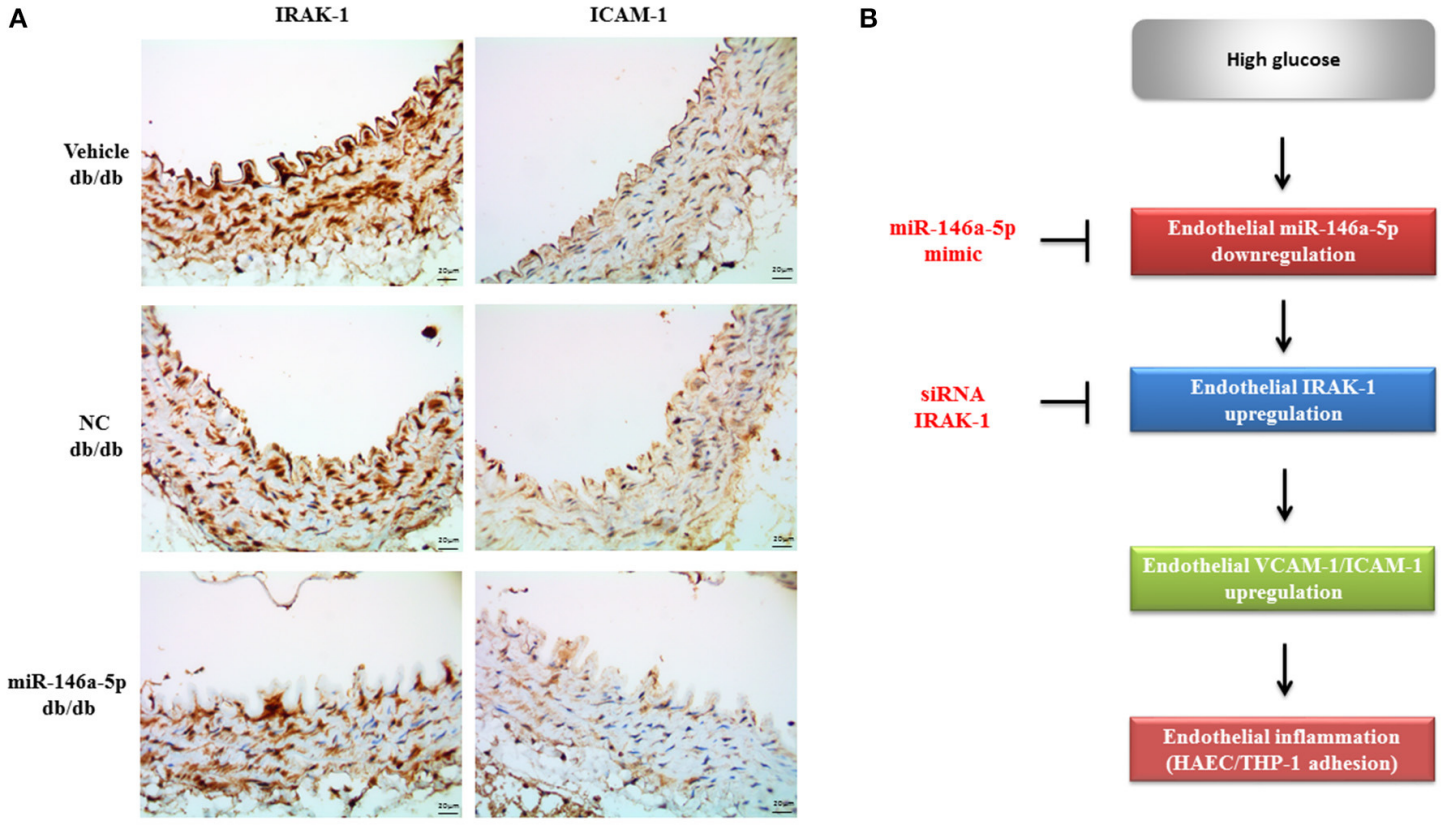
**(A)** The miR-146a-5p mimic decreased endothelial IRAK-1 and ICAM-1 expression in type 2 db/db diabetic mice. Immunohistochemistry of IRAK-1 and ICAM-1 in thoracic aorta tissue. Representative images showed that the immunoreactivities (brown color) of IRAK-1 and ICAM-1 in endothelial cells were dramatically decreased in the miR-146a-5p mimic-treated diabetic mice, compared to the negative control (NC) or vehicle-treated mice. *n* = 3 per group. Scale bar = 20 μm. **(B)** The proposed role of miR-146a-5p in regulating HG-induced endothelial inflammation via IRAK-1.

To examine the effect of the miR-146a-5p mimic on the expression of endothelial IRAK-1 and ICAM-1 *in vivo*, we performed IHC on aortic tissues from db/db type 2 diabetic mice. As shown in Figure 5A, aortic endothelial IRAK-1 and ICAM-1 protein levels from miR-146a-5p mimic-treated mice were dramatically decreased, as compared to negative control or vehicle-treated mice. This demonstrates that miR-146a-5p may have therapeutic potential, mitigating endothelial dysfunction through the downregulation of both IRAK-1 and ICAM-1. The proposed role of miR-146a-5p in regulating HG-induced endothelial inflammation via IRAK-1 is shown in Figure 5B.

## Discussion

This study demonstrates that HG induced IRAK-1 expression and endothelial inflammation in HAECs via the downregulation of miR-146a-5p expression. Among multiple mechanisms responsible for HG-induced endothelial inflammation, the induction of IRAK-1 plays a proinflammatory role in HG-stimulated HAECs, and represents an important mediator that maintains chronic inflammation in diabetic vascular diseases. Both *in vitro* and *in vivo* experiments identified miR-146a-5p as a target in treating diabetic vascular complications.

Among the different target prediction tools, the miRanda-mirSVR database possesses the advantageous properties of being easy to use, containing relatively up-to-date information, and possessing a large range of capacity (Peterson et al., 2014). Target genes can be predicted with miRanda by considering seed region-weighted algorithms, free energy analyses, and cross-species sequence conservation (Enright et al., 2003). In addition, the pre-computed mirSVR scores are useful in representing the effects of a specific miR on gene expression (Betel et al., 2010). Generally, a higher absolute value of mirSVR score indicates greater downregulation at the mRNA or protein levels. In our study, although miR-339-5p and miR-874-3p had mirSVR scores that ranked them fourth and fifth among our 13 miRanda-predicted miRs that would interact with the 3′-UTR of IRAK-1 mRNA, in the transfection assays, these miRs did not regulate IRAK-1 expression. Interestingly, miR-146a-5p had the highest pre-computed mirSVR score among the 13 predicated miRs. Furthermore, the 3′-UTR of IRAK-1 possessed two miR-146a-5p 7-mer seed region binding sites; these findings support the hypothesis that the interactions of the miR-146a-5p::IRAK-1 duplex are functional (Brennecke et al., 2005).

The role of IRAK-1 in diabetes is not clear. Recent studies have documented the involvement of TLR signaling in the metabolic aberrations of diabetes. In human microvascular endothelial cells incubated with HG, the expression levels of TLR4, MyD88, and IL-1β were increased (Wang et al., 2015), implying that both TLR-signaling and IL-1R-signaling are activated. In the monocytes of type 1 (Devaraj et al., 2008) and type 2 (Dasu et al., 2010) diabetes patients, levels of TLR2, TLR4, and other TLR-signaling components (e.g., MyoD88 and NF-κB) were increased. Our data demonstrated that HG induced the expression of an essential TLR/IL-1R signaling component: IRAK-1. Importantly, the increase in IRAK-1 expression was involved in the regulation of downstream endothelial inflammatory phenotypes, as HG-enhanced VCAM-1/ICAM-1 gene expression and monocyte adhesion was partially reduced after IRAK-1 depletion by siRNA.

In LPS-stimulated human monocytes, miR-146a-5p was identified as a negative regulator of the NF-κB pathway, targeting IRAK-1 and TRAF-6 expression (Taganov et al., 2006). In addition, several groups have reported that IL-1β, TNF-α, IL-8, and ox-LDL could stimulate increased expression of miR-146a-5p in different cell types (Chen et al., 2011; Li et al., 2012; Cheng et al., 2013). The miR-146a-5p promoter contains two NF-κB binding sites; these were responsible for the LPS/IL-1β/TNF-α-stimulated expression of miR-146a-5p (Taganov et al., 2006). Although the activation of endothelial NF-κB by HG has been reported (Ho et al., 2006), HG did not induce endothelial miR-146a-5p expression. In our previous work, we found that in HAECs, a 5 h HG-stimulation could downregulate miR-146a-5p expression to 83% of that observed in the control (Wang, H. J. et al., 2014). We also reported that glycated albumin can downregulate endothelial miR-146a-5p expression (Wang et al., 2013). Different mouse tissues, including retina, heart, kidney, diabetic wound, and dorsal root ganglion neuron, also displayed decreased expression of miR-146a-5p, suggesting that diabetes-associated injuries, including those due to hyperglycemia, contributed to the prolonged reduction of miR-146a-5p expression observed *in vivo* (Feng et al., 2011, 2017; Xu et al., 2012; Wang, L. et al., 2014). In this study, we extended HG stimulation of HAECs for 24 and 48 h, and the results display sustained downregulation of miR-146a-5p, to 18 and 68% of the control levels, respectively. At 24 and 48 h, VCAM-1/ICAM-1 gene expression levels and THP-1 adhesion were enhanced, suggesting that the role of miR-146a-5p as an anti-inflammatory brake had been impaired.

Although the 3′-UTRs of VCAM-1 and ICAM-1 mRNA are not predicted to bind to miR-146a-5p, we observed that HG-induced VCAM-1 and ICAM-1 expression levels and THP-1 adhesion were effectively inhibited by the miR-146a-5p mimic. These results suggest that the expression of VCAM-1 and ICAM-1 is indirectly regulated by miR-146a-5p in HG-stimulated HAECs. Both the VCAM-1 and ICAM-1 promoters require NF-κB for maximal levels of induction (Collins et al., 1995); therefore, the inhibitory effects of the miR-146a-5p mimic were most likely mediated through NF-κB. Apart from the indirect regulation of inflammatory molecules through the NF-κB pathway, miR-146a-5p has multiple direct targets that modulate different inflammatory pathways. At the receptor level, TLR-4 is a direct miR-146a-5p target in oxidized low-density lipoprotein-stimulated macrophages (Yang et al., 2011). Downstream from TLR-4, both IRAK-1 and TRAF-6 are known miR-146a-5p targets that dampen LPS-induced inflammation in monocytes (Taganov et al., 2006). NADPH oxidase 4 (NOX4) is an important mediator responsible for diabetic complications, and our previous work revealed that miR-146a-5p was a direct regulator of NOX4 in HG/thrombin-stimulated HAECs (Wang, H. J. et al., 2014). In gastric cancer, in addition to its role in TLR/IL-1R signaling, miR-146a-5p has been reported to directly control the G-protein-coupled receptor-mediated activation of NF-κB, via caspase recruitment domain-containing protein 10 (CARD10) and COP9 signalosome complex subunit 8 (COPS8) (Crone et al., 2012). Overall, multiple inflammatory mediators are regulated by miR-146a-5p, emphasizing the evolutionary effectiveness of restraining excessive inflammation via a single mediator.

## Author contributions

HW, CP, and WL conceived the project. HW and WL wrote the manuscript. HW, CP, and WL provided funding. HW and WL performed critical experiments. WL and HW supervised the study.

### Conflict of interest statement

The authors declare that the research was conducted in the absence of any commercial or financial relationships that could be construed as a potential conflict of interest.
