# Supplementary material for: MicroRNA-146a-5p Mediates High Glucose-Induced Endothelial Inflammation via Targeting Interleukin-1 Receptor-Associated Kinase 1 Expression
Source: Front Physiol. 2017 Aug 2;8:551. doi: 10.3389/fphys.2017.00551 (PMC5539227; doi:10.3389/fphys.2017.00551)
Supplement: Supplementary file 1 [file DataSheet1.DOCX]

**Title: MicroRNA-146a-5p Mediates High Glucose-Induced Endothelial Inflammation via Targeting Interleukin-1 Receptor-Associated Kinase 1 Expression**

**Authors: Wan-Yu Lo, PhD; Ching-Tien Peng, MD; Huang-Joe Wang*, MD, PhD**

| **Supplement Data 1. Primer set list for mRNAs and microRNAs** | |
| --- | --- |
| **GAPDH** | Forward primer: 5'-CTCTGCTCCTCCTGTTCGAC-3'  Reverse primer: 5'-ACGACCAAATCCGTTGACTC-3' |
| **IRAK-1** | Forward primer: 5’-GAGACCTTGGCTGGTCAGAG-3’  Reverse primer: 5’-GTGCTTCTCAAAGCCACTCC-3’ |
| **VCAM-1** | Forward primer: 5’-TGCACAGTGACTTGTGGACAT-3’  Reverse primer: 5’-CCACTCATCTCGATTTCTGGA-3’ |
| **ICAM-1** | Forward primer: 5’-CCTTCCTCACCGTGTACTGG-3’  Reverse primer: 5’-AGCGTAGGGTAAGGTTCTTGC-3’ |
| **RNU6B** | Reverse transcription primer:  5'-GTTGGCTCTGGTGCAGGGTCCGAGGTATTCGCACCAGAGCCAACAAAAATAT-3'  Forward primer: 5'-TTCCTCCGCAAGGATGACACGC-3'  Reverse primer: 5'-GTGCAGGGTCCGAGGT-3' |
| **miR-146a-5p** | Reverse transcription primer:  5'-GTTGGCTCTGGTGCAGGGTCCGAGGTATTCGCACCAGAGCCAACAACCCA-3'  Forward primer: 5'-CCGCCGTGAGAACTGAATTCCA-3'  Reverse primer: 5'-GTGCAGGGTCCGAGGT-3' |
| **miR-339-5p** | Reverse transcription primer:  5’-GTTGGCTCTGGTGCAGGGTCCGAGGTATTCGCACCAGAGCCAAC CGTGAG-3’  Forward primer: 5’-TTGTCCCTGTCCTCCAGG-3’  Reverse primer: 5’-GTGCAGGGTCCGAGGT-3’ |
| **miR-874-3p** | Reverse transcription primer:  5’-GTTGGCTCTGGTGCAGGGTCCGAGGTATTCGCACCAGAGCCAACTCGGTC-3’  Forward primer: 5’-TTTTTCTGCCCTGGCCCG-3’  Reverse primer: 5’-GTGCAGGGTCCGAGGT-3’ |
| **miR-125-3p** | Reverse transcription primer:  5’-GTTGGCTCTGGTGCAGGGTCCGAGGTATTCGCACCAGAGCCAACGGCTCC-3’  Forward primer: 5’-GTGACAGGTGAGGTTCTTG-3’  Reverse primer: 5’-GTGCAGGGTCCGAGGT-3’ |
| **miR-431-5p** | Reverse transcription primer:  5’-GTTGGCTCTGGTGCAGGGTCCGAGGTATTCGCACCAGAGCCAACTGCATG-3’  Forward primer: 5’-GTTTTGTCTTGCAGGCCGT-3’  Reverse primer: 5’-GTGCAGGGTCCGAGGT-3’ |
| **miR-192-5p** | Reverse transcription primer:  5’-GTTGGCTCTGGTGCAGGGTCCGAGGTATTCGCACCAGAGCCAACGGCTGT-3’  Forward primer: 5’-GGGGCTGACCTATGAATTG-3’  Reverse primer: 5’-GTGCAGGGTCCGAGGT-3’ |
| **miR-215-5p** | Reverse transcription primer:  5’-GTTGGCTCTGGTGCAGGGTCCGAGGTATTCGCACCAGAGCCAACGTCTGT-3’  Forward primer: 5’-GTTTGGGATGACCTATGAATTG-3’  Reverse primer: 5’-GTGCAGGGTCCGAGGT-3’ |

| **Supplement Data 2. ON-TARGETplus Human IRAK1 siRNA – SMARTpool** |
| --- |
| ON-TARGETplus SMARTpool siRNA J-004760-12, IRAK1  Target Sequence: GAUGAGAGGCUGACACCCA |
| ON-TARGETplus SMARTpool siRNA J-004760-13, IRAK1  Target Sequence: CGAAGAAAGUGAUGAAUUU |
| ON-TARGETplus SMARTpool siRNA J-004760-14, IRAK1  Target Sequence: GCAAUUCAGUUUCUACAUC |
| ON-TARGETplus SMARTpool siRNA J-004760-15, IRAK1  Target Sequence: GAGCUGAUGUGUUCACCUG |

**Supplemental Data 3.**(A). Endothelial IRAK-1 expression was not increased by mannitol. n=3, ** *p* < 0.01. ****p*<0.001. N.S., not significant. (ANOVA) (B). Endothelial miR-146a-5p expression was not increased by mannitol. n=3, ****p*<0.001. N.S., not significant. (ANOVA)


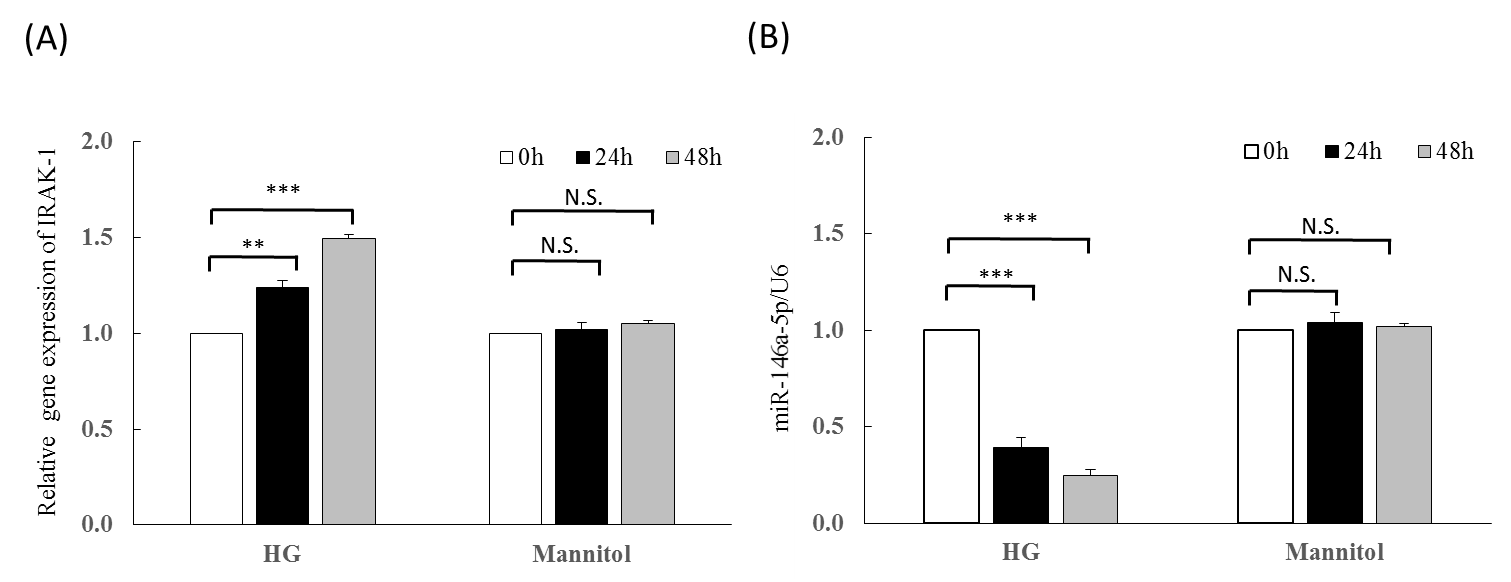


| **Supplemental Data 4. Predicated miRNAs target IRAK-1** | |
| --- | --- |
| MicroRNA name | Absolute value of mirSVR scores |
| miR-146a-5p | 1.6713 |
| miR-146b-5p | 1.6713 |
| miR-346 | 0.6294 |
| miR-339-5p | 0.5597 |
| miR-874-3p | 0.442 |
| miR-125-3p | 0.3878 |
| miR-150-5p | 0.3616 |
| miR-490-3p | 0.2421 |
| miR-431-5p | 0.1949 |
| miR-142-3p | 0.1851 |
| miR-485-5p | 0.162 |
| miR-192-5p | 0.1081 |
| miR-215-5p | 0.1081 |
